# Reviving Iran’s Science Diplomacy: New Horizons Opened by President Pezeshkian

**DOI:** 10.34172/aim.33115

**Published:** 2025-01-01

**Authors:** Farid Rahimi, Mohammad Javad Zarif, Amin Talebi Bezmin Abadi

**Affiliations:** ^1^Research School of Biology, The Australian National University, Ngunnawal and Ngambri Country, Canberra, ACT, Australia; ^2^Department of Global Studies, Faculty of World Studies, University of Tehran, Tehran, Iran; ^3^Department of Bacteriology, Faculty of Medical Sciences, Tarbiat Modares University, Tehran, Iran

 Outcomes of well-grounded basic or applied sciences benefit societies, the environment, and life on Earth. Scientific enterprise and progress require time, effort, dedication, training, technology, supplies, logistics, and specialized human resources. Academics and scientists serve their communities by producing papers or patents to test, prove, or debate hypotheses; by facilitating or guiding public debate and policy; contributing to undergraduate or postgraduate teaching by developing and delivering curricula; or by peer-reviewing, consultation, and mentoring. Meanwhile, they cultivate national or international academic and scholarly collaborations by science diplomacy. Thus, Iranian researchers are indeed science diplomats as they publish in prestigious journals, serve as members of international editorial boards, or participate in international workshops or conferences.

 While research and scientific enterprise are valued and supported in the developing countries,^1^ Iranian academics and scholars are also keen to produce and showcase their sophisticated studies and contribute globally. The quality and quantity of publications generally reflect the scientific reputation and academic standing of a nation. Almost twelve years ago, Iran had the world’s fastest-growing scientific output, more so than that of China.^2^ Contrarily, the number of Iran-affiliated papers plummeted to 74 220 (Scopus data) for the first time in the past three years, with 2022 capturing the highest. This reduced productivity may likely represent Iran’s weakened science diplomacy within the region or internationally, or may reflect ignorance to, or neglect of, the importance of such statistics by the policymakers and relevant stakeholders.

 However, overall, the Iranian academics have achieved highly impressive ranking and statistics during the 44 years of sanctions imposed by the United States, which isolated the Iranian scientific community.^3^ Ironically, Iran captured the most collaborations with the United States before and during the sanctions (55 366 documents on Scopus since 1818). Many Iran-affiliated scientific publications feature collaborations with other Western countries, more tangibly with Canada, the United Kingdom, Australia, and Germany (Scopus data). Owing to the unstable relations between Iran and the United States in the past decades, using any approach to improve the level of collaborations is difficult, including science diplomacy. However, prioritizing the removal of unilateral economic sanctions alongside with widening international collaborations can directly boost science diplomacy by Iran. While many Western countries led by the United States’ government removed their long-lasting sanctions on essential drugs, medical equipment, and matters related to academia, Iranian scholars are still affected by other sanctions coupled with the general hesitation to publish Iranian papers. These challenges need to be thoroughly assessed and redressed by the relevant policymakers and authorities.

 The election success of Masoud Pezeshkian on July 6, 2024 was welcomed by Iranian scholars. While some Iranian–American scholars are less hopeful of the revival of Iranian scientific collaborations,^4^ President Pezeshkian has vowed to change the situation. Pezeshkian promises to improve Iran’s economy by engaging directly with the Western countries and by working to lift sanctions through negotiations focusing on the revival of the Joint Comprehensive Plan of Action ( JCPOA). Western states are encouraged to reduce their sanctions’ pressures and collaborate by contributing to the Iranian academic ventures. In such circumstances, invigorating science diplomacy is an efficient alleviating factor. Thus, the political mindset will need to change about international collaborations. Accordingly, some regional agreements, for example, between Saudi Arabia and Iran, will nurture scientific and research collaborations irrespective of disputable or disputed political interests (https://www.reuters.com/world/middle-east/iran-saudi-arabia-agree-resume-ties-re-open-embassies-iranian-state-media-2023-03-10/). In parallel, revival of JCPOA or any alternative (https://www.brookings.edu/articles/reviving-the-jcpoa-is-the-better-alternative-but-can-it-be-made-sustainable/) facilitates trade between Iran, its global partners, and its neighbors. Thus, importing scientific supplies, technologies, instruments, and consumables will equip the Iranian research institutions and clinical laboratories competitively, boosting academic and medical capabilities. Additionally, JCPOA will ease international banking transactions enabling authors to pay Article Processing Charges internationally.

 We believe three factors can revive, amend, and boost science diplomacy by Iran:

- Establishing multiple agreements between Iranian and Western universities will garner long-term, bilateral, mutually beneficial academic projects.
- Encouraging co-authorships and mutual participation of Iranian and international scientists in editorial boards of highly ranked scientific journals will increase international academic links and collaborations.
- Agreements to freely use subscription-based access to the electronic libraries across the world is another example of mutual collaborations. Holding international conferences in Tehran jointly with other Western capitals without restricting financial transactions online may also foster science diplomacy particularly regarding the research fields or topics of mutual interest.
